# Chinese parents' willingness to vaccinate their children against COVID-19: A systematic review and meta-analysis

**DOI:** 10.3389/fpubh.2022.1087295

**Published:** 2022-12-15

**Authors:** Yundi Ma, Jingjing Ren, Yang Zheng, Dongping Cai, Shuai Li, Yangni Li

**Affiliations:** ^1^Department of General Practice, The First Affiliated Hospital, Zhejiang University School of Medicine, Hangzhou, Zhejiang, China; ^2^The Healthcare Center for Shishan Street Community of Suzhou New District, Suzhou, Jiangsu, China

**Keywords:** Chinese parents, children, COVID-19 vaccine, predictors, systematic review, vaccination willingness

## Abstract

**Introduction:**

To evaluate Chinese parents' willingness to vaccinate their children against COVID-19, identify its predictors, and provide a reference for raising the COVID-19 vaccination rate for children.

**Method:**

PubMed, Cochrane Library, Embase, and the databases in Chinese, including CNKI, WanFang, VIP, CBM, were searched from December 2019 to June 2022, and citation tracking was used to identify relevant studies. To calculate the rate with 95% confidence intervals (CI), a random-effects model was used. To explore sources of heterogeneity, sensitivity analysis and subgroup analysis were conducted. This analysis was registered on PROSPERO (CRD42022346866) and reported in compliance with the PRISMA guidelines.

**Result:**

Overall, 80 studies were screened, and 13 studies with 47994 parents were included after removing duplicates and excluding 19 studies that did not meet the selection criteria by title, abstract and full-text screening. The pooled willingness rate of Chinese parents to vaccinate their children against COVID-19 was 70.0% (95% CI: 62.0~78.0%). Level of education, perceived susceptibility of children infected with COVID-19, and parental attitudes toward vaccination (such as perceived efficacy and safety of the COVID-19 vaccines, parental willingness to vaccinate themselves, parental vaccination hesitancy, and the history of children's vaccination against influenza) were the main predictors of parents' intention to vaccinate their children.

**Discussion:**

Chinese parents' willingness to vaccinate their children against COVID-19 is moderate, and factors including parental education level, perceived susceptibility of children infected with COVID-19, and parental attitudes toward vaccination affect this decision. Fully identifying these factors and their mechanism will be essential to further raise the willingness rate.

**Systematic review registration:**

https://www.crd.york.ac.uk/PROSPERO/, identifier: CRD42022346866.

## Introduction

The COVID-19 epidemic was caused by severe acute respiratory syndrome coronavirus 2 (SARS-CoV-2), which was mainly transmitted through direct transmission, contact transmission, and airborne transmissions, such as cough, sneeze, droplet inhalation, contact with oral, nasal and eye mucous membranes and other common modes of spread (1). And the COVID-19 epidemic started from Wuhan city of China toward the end of December 2019 and since then has been spreading globally (1). As of June 27, 2022, the WHO has received reports of approximately 540 million confirmed COVID-19 cases and nearly 6.32 million deaths^1^. Among the infected individuals in the United States, children made up 14.3%. Compared to adults, children infected with SARS-CoV-2 often exhibit milder or silent clinical symptoms, but there are still a small number of severe infections that can result in hospitalization or even death (2). In addition, because the symptoms of COVID-19 infection in children are indistinguishable from those of other respiratory infections, which are the most common in children, these may lead to the untimely treatment of infected children and may increase the spread of infection within the community (including homes, child care centers, and schools) (2).

Although strict measures in modern times have been implemented to curb the spread of the virus, including mask wearing, social distancing, stay-at-home orders, restrictions on travel and gatherings, and closures of schools and businesses, the COVID-19 epidemic is still not fully under control at home or abroad (3). In addition, the above measures have created social isolation, which may undermine the healthy physical and mental development of children (4). Although there are remdesivir, monoclonal antibodies and other drugs against the COVID-19 virus, they cannot be promoted due to some factors (5). Due to its controversial clinical efficacy for remdesivir is that the drug was mainly tested for treatment of severe COVID-19 patients in which antiviral drugs may not be very useful; Monoclonal antibodies such as Bamlanivimab and etesevimab, and Sotrovimab have already been successfully developed and authorized for use in patients with mild COVID-19 and high risk factors, but their use cannot be promoted due to high price tag, limitations in large-scale production, and vulnerability to virus variants (5, 6). Therefore, the most effective and cost-effective way to prevent infectious diseases is vaccination (7). In addition to the benefits to individuals, vaccinating children against COVID-19 will help to succeed in achieving substantial control of community spread, without the disruptions of society caused by COVID-19 over the past 2 years (8). Moreover, several studies have shown that the COVID-19 vaccine has good safety and efficacy in children, and the United States began vaccinating children and adolescents 12 years of age and older with the COVID-19 vaccine in May 2021 (9). In July 2021, China was also actively conducting research, developing specific policies, and legalizing the emergency use of inactivated SARS-CoV-2 vaccine in children and adolescents aged 3 to 17 years (2).

Although many countries try hard to develop a vaccine for COVID-19, the success of vaccination programs also require high rates of public acceptance and population coverage (10). In terms of public vaccination acceptance, studies have reported that low rates of public acceptance or vaccine hesitancy may prolong pandemics, increase mortality and infection rates, and put more pressure on health systems (11, 12). Previous studies already predicted that vaccine hesitancy could be a significant challenge for COVID-19 vaccine rollout (13, 14). In addition, some prior literature studied in China, India, Canada, the United States and other countries and regions have published on COVID-19 vaccine hesitancy or willingness demonstrating that the factors that are responsible for vaccine hesitancy or willingness range from social demographics, occupation, religious beliefs, and social and environmental trust (15–18). In these studies, parents were more likely to be hesitant to vaccinate if they were female, younger, more concerned about the safety and efficiency of the vaccine, reluctant to vaccinate themselves, unaware of the risk of COVID-19 infection for themselves and their children, and distrustful of the social environment (16–18).

And parents, as guardians of their minor children, have the right to decide on their children's COVID-19 vaccination. Hence, it is crucial to understand parents' willingness to vaccinate their children against COVID-19 and above associated predictors (19). Previous literature has systematically evaluated the willingness of parents to vaccinate their children against COVID-19 and the factors influencing it in the United States, China, Italy, the United Kingdom, India, and other countries and regions (17, 18). To our knowledge, no systematic national and subregional descriptions of Chinese parents' willingness to vaccinate their children with COVID-19 alone have been conducted previously. Therefore, this study aimed to systematically estimate Chinese parents' willingness to vaccinate their children against COVID-19 nationally and regionally and to identify predictors of vaccine willingness or vaccine hesitancy to promote improved vaccination rates and achieve substantial control of community spread in the future.

## Materials and methods

This systematic review and meta-analysis was conducted in compliance with the Preferred Reporting Items for Systematic Reviews and Meta-Analyses (PRISMA) guidelines, and the protocol was registered on PROSPERO (CRD42022346866).

### Search strategy

Two authors (YM and YZ) respectively searched PubMed, Cochrane Library, Embase, and the databases in Chinese, including CNKI, WanFang, VIP, CBM, from December 2019 to June 2022. Both controlled terms (e.g., MeSH terms in PubMed) and free-text terms were used based on the following topics and their synonyms: parents, legal guardians, COVID-19, SARS-CoV-2, COVID-19 vaccines, willingness, intention, vaccination hesitancy, vaccination refusal, acceptance, China, and Chinese. The search string is shown in Appendix 1 in detail. All related published papers were stored using EndNote (version, X9.2 (Bld 13018), developed by Clarivate Analytics).

### Eligibility criteria

We aimed to systematically estimate Chinese parents' willingness to vaccinate their children against COVID-19 nationally and regionally and to identify predictors of vaccine willingness or vaccine hesitancy. Thus, original records were selected based on the following inclusion criteria: (1) studies involving Chinese parents' willingness to vaccinate their children against COVID-19; (2) the target population was adult participants (>18 years) with children aged 3 to 17 years in China (based on China's vaccination policy for children); (3) studies providing specific survey data for pooling; (4) published in English and/or Chinese; and (5) cross-sectional study.

The exclusion criteria were as follows: (1) non-primary studies with no initial specific survey data available: reviews, case reports, editorials, systematic reviews, and other non-primary articles; (2) studies with only subgroup-specific samples (e.g., health care workers or patients); and (3) duplicate studies or databases.

### Data extraction

Two authors (YM and YZ) screened all of the sources for inclusion, with a third senior author (DC) consulted when disagreement occurred. After eliminating duplicates, two independent authors (YM and YZ) screened titles and abstracts and then used predefined criteria to screen the full text of potentially relevant articles. Data from eligible studies were extracted into a database constructed with Microsoft Excel 2019. We extracted the following information from the included articles: title, first author, study type, study location, study area, recruitment method, study collection date, sampling method, survey method, questionnaire response rate, sample size, number of willing participants, gender of parents, age of parents and children, level of education, marital status, type of occupation, and parental attitudes toward vaccination, including perceived efficacy and safety of the COVID-19 vaccines, parental willingness to vaccinate themselves, parental vaccination hesitancy, and the history of children's vaccination against influenza (details in Appendix 2).

### Quality assessment

The quality of each study was assessed using the modified Newcastle–Ottawa Scale (NOS) for cross-sectional studies (20). The NOS assesses three domains of methodology of study, including study participant selection (0–5 points), confounder adjustment (0–2 points), and outcome indicator determination (0–3 points) (21). Studies scoring ≥5 out of 10 points were included in the present systematic review (22).

### Data analysis

To calculate the rate with 95% confidence intervals (CI), the metaprop command in Stata software (version MP 17.0, developed by StataCorp, Inc.) was conducted. And the Freeman–Tukey double arcsine transformation of the original willingness rate was conducted to stabilize the variance to reduce the effect of extreme values on the pooled willingness rate estimate (23). We used the Hedges Q statistics and *I*^2^ to assess heterogeneity between studies, with *I*^2^ ≥ 50% considered to be significant heterogeneity. We applied a random effect model to estimate pooled effects since the heterogeneity between results was significant. Subgroup analyses were conducted to explore the sources of heterogeneity. A stratified analysis of factors influencing parents to vaccinate their children with COVID-19 was performed using Rstudio (version 1.4.1106) to avoid confounding bias.

We conducted a leave-one-out sensitivity analysis to determine the influence of each study on the overall effect (18). We used the funnel plot and Egger's test to assess publication bias. A *P*-value < 0.05 indicates statistical significance. And statistical significance is important because it may be said to measure the reliability of the results, that is, the probability of getting the same results if the studies were repeated and the test of statistical significance provides a measure of the likelihood that the differences among outcomes are actual, and not just due to chance. It is also for these reasons that it allows researchers to hold a degree of confidence that their findings are real and reliable and not due to chance (24, 25).

## Results

**Figure 1:**
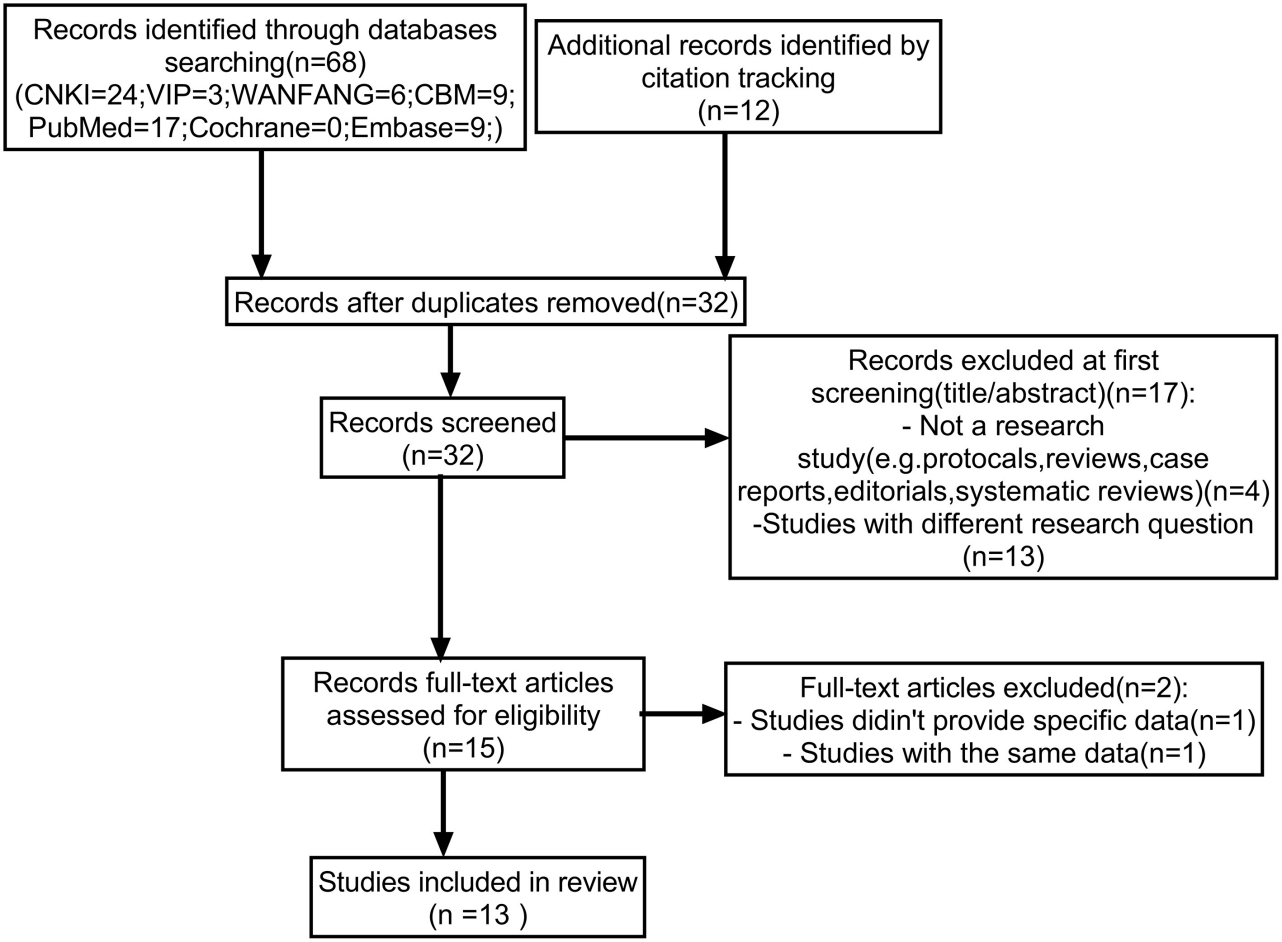
Flowchart diagram of the study selection for systematic review.

Overall, 80 studies were screened from the database and by citation tracking. After removing 48 duplicates and excluding 17 studies that did not meet the selection criteria by title or abstract screening, a total of 15 studies were assessed for full text, of which two studies were further excluded because specific data could not be extracted (*n* = 1) and duplicated data (*n* = 1). Finally, 13 studies with 47,994 parents were included (Figure 1).

Details of the studies included in this systematic review are presented in Table 1. Data were collected from February 2020 to November 2021. The sample size ranged from 468 to 13,451 parents, with a median of 3,692 parents. Three studies were conducted in Shenzhen; two studies in Shanghai; two studies nationwide; and one study each in Qinghai, Taizhou, Wuxi, Kaifeng, and Wuhu. Additionally, one study compared parents' willingness to vaccinate their children against COVID-19 in Zhejiang and Shandong. Nine studies used a convenience sampling method, and four studies used a random sampling method. In five studies, parents were recruited *via* online surveys or their children's schools. In six studies, parents were recruited in a clinical environment, such as a physical examination center, an immunization clinic, a local community health care center, or other clinical settings. In the other two studies, parents were recruited by both of these methods. Eight studies did not report the response rate. The quality assessment of the cross-sectional studies included in this systematic review is shown in Supplementary Table 1.

**Table 1:** Characteristics of included studies.

**Reference**	**Study location**	**Study area**	Recruitment method^*^	**Survey method**	**Sampling method**	**Response rate (%)**	**Sample size (*n*)**	**Vaccination willing (*n*)**	**Quality score**
Zhang et al. (19)	Shenzhen	Eastern region	Hospital	Online	Convenience	77.4	1,052	764	8
Kezhong et al. (26)	Qinghai	Western region	Hospital	Online	Convenience	NR	13,451	6,723	7
Li et al. (14)	Shenzhen	Eastern region	Hospital	Online	Convenience	NR	3,342	2,976	7
Zhang et al. (27)	Taizhou	Eastern region	Non-hospital	Online	Convenience	72.6	1,788	831	8
Wang et al. (28)	Wuxi	Eastern region	Hospital	Offline	Stratified random	NR	3,009	1,784	8
Wan et al. (7)	Kaifeng	Central region	Non-hospital	Offline	Two-stage stratified random	100	468	406	8
Yang et al. (29)	China	\	Non-hospital	Online	Convenience	NR	12,872	9,122	7
Xu et al. (30)	Shenzhen	Eastern region	Non-hospital	Online	Convenience	97.12	4,748	3,451	8
Lin et al. (15)	China	\	Non-hospital	Online	Convenience	NR	2,026	1,573	7
Yunyun et al. (31)	Shandong, Zhejiang	Eastern region	Hospital	Online	Random	NR	917	773	7
Zhou et al. (3)	Shanghai	Eastern region	Hospital	Online	Convenience	NR	747	637	7
Wu et al. (32)	Shanghai	Eastern region	Hospital and non-hospital	Online	Stratified cluster random	99.37	2,538	1,499	9
Wu et al. (33)	Wuhu	Central region	Hospital and non-hospital	Online	Convenience	NR	1,036	453	7

* Recruitment method: Hospital refers to recruiting parents in a clinical setting, such as a physical examination center, an immunization clinic, a local community health care center, or other clinical settings; Non-hospital refers to recruiting parents via their children's schools or online surveys, such as the WeChat-incorporated, Wen Juan Xing platform and other online surveys.

NR: not reported.

**Figure 2:**
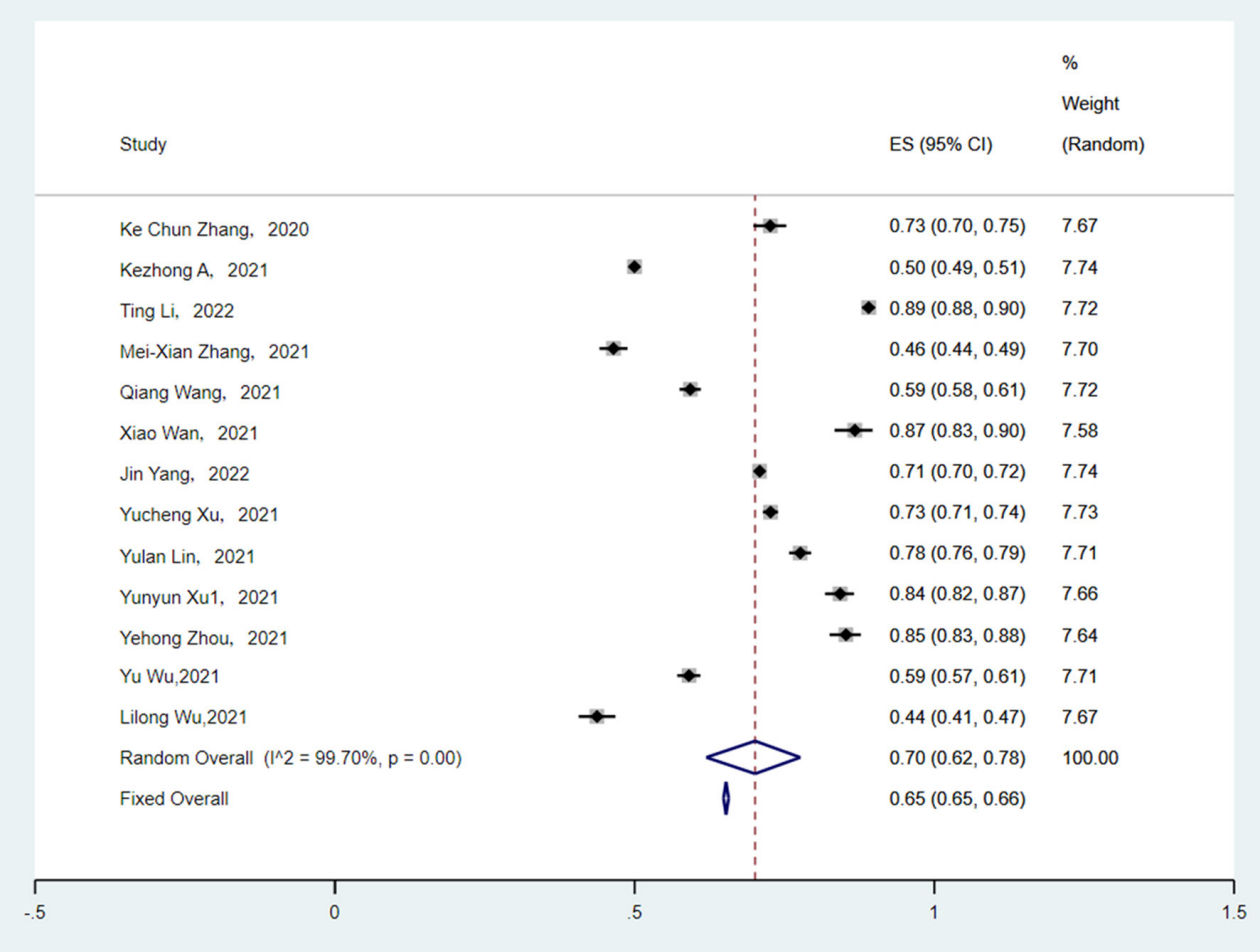
Forest plot.

Thirteen studies reported the number of parents who were willing to vaccinate their children. The heterogeneity between the results was very high (*I*^2^ = 99.70%, *p* < 0.001). The random effect model was applied to estimate pooled effects. The pooled rate of parents who intended to vaccinate their children against COVID-19 was 70.0% (95% CI: 62.0–78.0%) (Figure 2). The parents' willingness rate ranged from 44.0 to 89.0%.

The results of subgroup analyses performed in Table 2 indicated that the parental willingness to vaccinate children varied across study areas (*p* < 0.001), significantly contributing to the heterogeneity. In the three regions of China, the majority of the study was conducted in the eastern region (8/13), with an estimated vaccination willingness rate of 71.0% (95% CI: 61.0–82.0%). The willingness rate was highest in the eastern region and lowest in the western region (50.0%, 95% CI: 49.0–51.0%). However, subgroups of the study collection date, age of parents, gender of parents, marital status, level of education, and health care-related occupations failed to explain the source of heterogeneity.

**Table 2:** The results of subgroup analyses.

**Subgroups**	**No of studies**	**Willingness Rate (%) (95% CI)**	***I*** **^2^ (%)**	***p*** **-value**
**Study collection date**				0.635
2020	7	70.0(60.0–79.0)	99.71	
2021	5	65.0(45.0–84.0)	99.80	
**Study area**				<0.001
Eastern region	8	71.0(61.0–82.0)	99.65	
Western region	1	50.0(49.0–51.0)	\	
Central region	2	65.0(63.0–67.0)	\	
**Age of parents**				0.952
≤ 40	5	69.0(57.0–80.0)	99.29	
>40	5	69.0(56.0–83.0)	98.08	
**Gender of parents**				0.679
Male	8	65.0(50.0–79.0)	99.38	
Female	8	61.0(49.0–72.0)	99.61	
**Marital status**				0.103
Two-parent family	3	57.0(27.0–88.0)	\	
Single-parent family	3	32.0(26.0–37.0)	\	
**Level of education**				0.428
High school and below	7	66.0(50.0–82.0)	99.48	
University and above	7	57.0(42.0–72.0)	99.64	
**Health care-related occupations**				0.942
Yes	5	69.0(55.0–82.0)	92.63	
No	5	69.0(58.0–81.0)	99.39	

**Figure 3:**
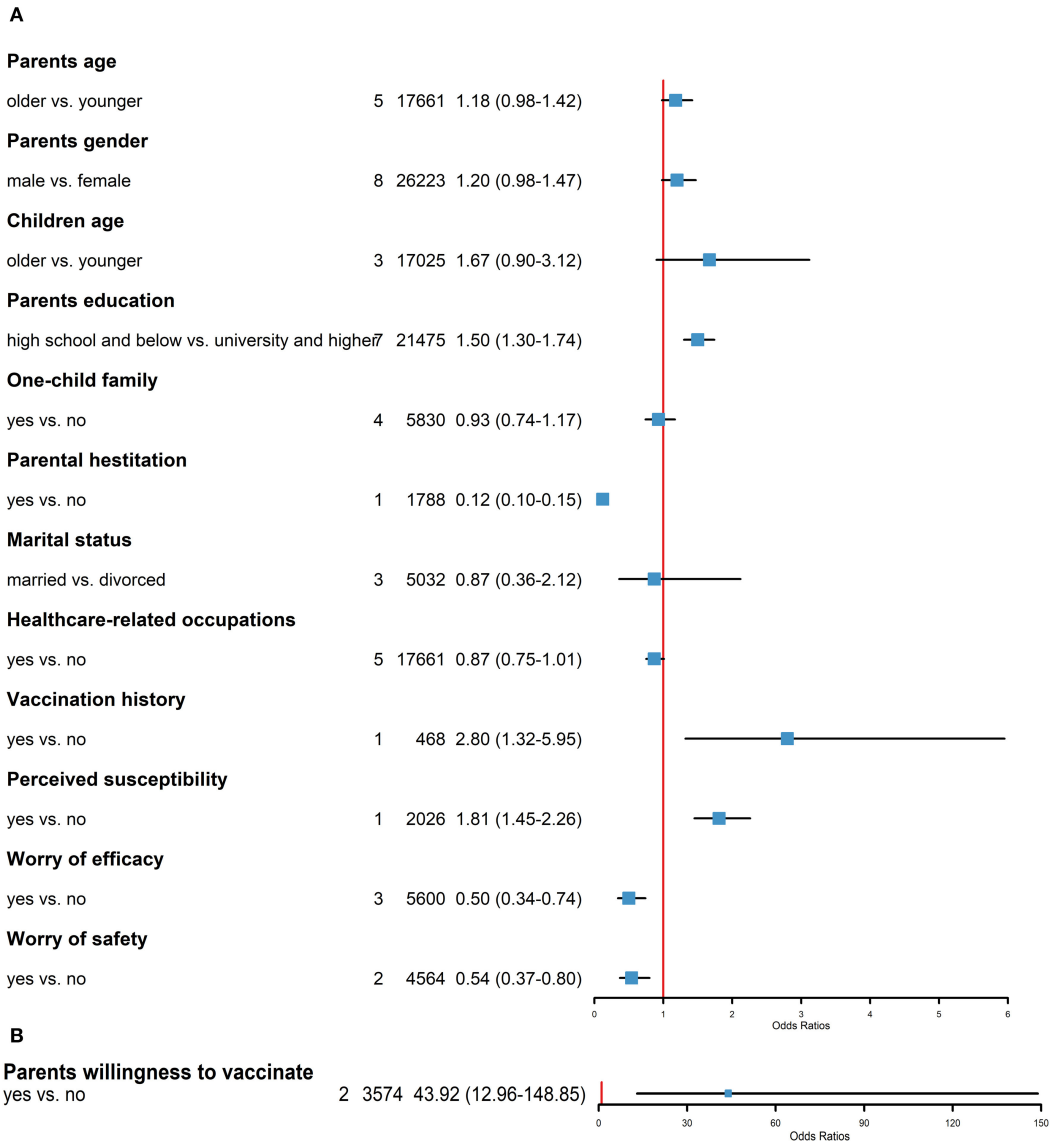
Predictors of Chinese parents' willingness to vaccinate their children against COVID-19.

Predictors of Chinese parents' willingness to vaccinate their children against COVID-19 were statistically analyzed, and the results are shown in Figure 3. The factors were divided into the following two main categories: (1) sociodemographic factors and (2) vaccination. Among the sociodemographic factors, parental gender (8/13), education level (7/13), parental age (5/13), and health care-related occupation (5/13) were the most frequent predictive factors reported in the included studies. Parents with education levels of high school and below were more likely to vaccinate their children against COVID-19 than those with education levels of university and above (OR: 1.50, 95% CI: 1.30–1.74). Parental age (OR: 1.18, 95% CI: 0.98–1.42), gender of parents (OR: 1.20, 95% CI: 0.98–1.47), children's age (OR: 1.67, 95% CI: 0.90–3.12), one-child family (OR: 0.93, 95% CI: 0.74–1.17), marital status (OR: 0.87, 95% CI: 0.36–2.12), and health care-related occupation (OR: 0.87, 95% CI: 0.75–1.01) were not statistically correlated with the willingness rate.

Among the vaccination factors, 13 studies reported the impact of vaccine drivers on parents' willingness or hesitancy to vaccinate their children against COVID-19. A history of children's vaccination against influenza (OR: 2.80, 95% CI: 1.32–5.95), parents' willingness to vaccinate against COVID-19 (OR: 43.92, 95% CI: 12.96–148.85), and perceived risk of COVID-19 infection in children (OR: 1.81, 95% CI: 1.45–2.26) were associated with increased parental willingness to vaccinate their children. In contrast, parental vaccination hesitancy (OR: 0.12, 95% CI: 0.10–0.15), concerns about the safety of vaccines (OR: 0.54, 95% CI: 0.37–0.80), and concerns about the efficacy of vaccines (OR: 0.50, 95% CI: 0.34–0.74) decreased parents' willingness to vaccinate their children.

**Figure 4:**
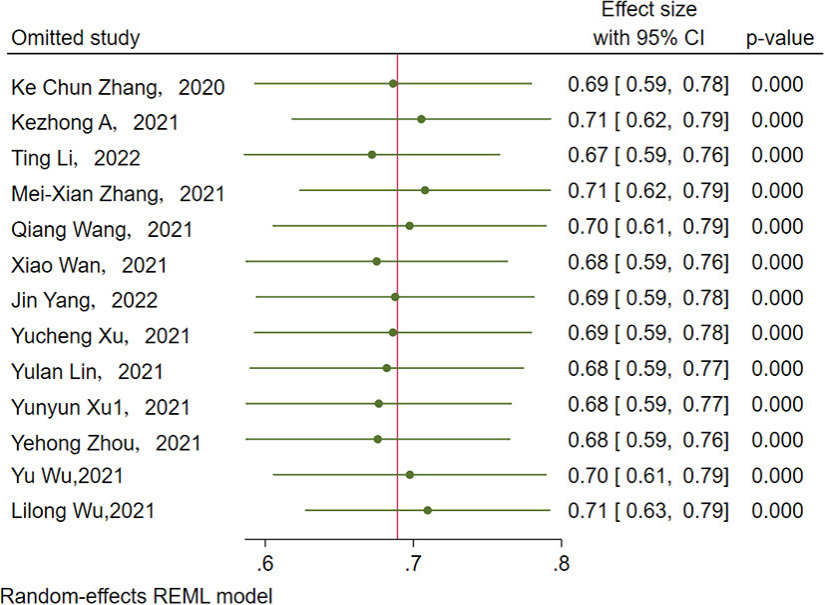
Forest plot of influential analysis on incidence studies.

**Figure 5:**
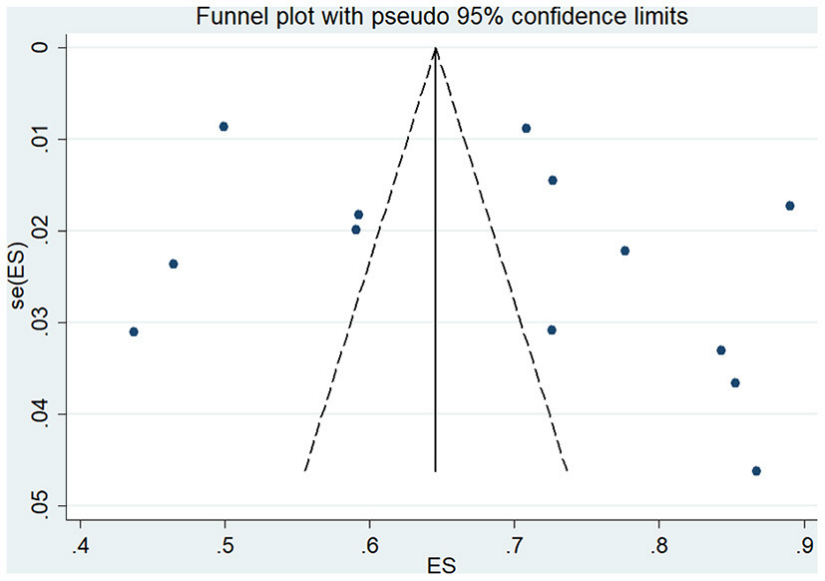
Funnel plot.

Sensitivity analysis showed that no single study had a disproportional effect on the pooled rate, which varied between 67.0% (95% CI: 59.0–76.0%) and 71.0% (95% CI: 62.0–79.0%) (Figure 4). A funnel plot and Egger's test were performed to assess the publication bias, and the results did not show evidence of publication bias (*p* > 0.05) (Figure 5).

## Discussion

SARS-CoV-2 was mainly transmitted through direct transmission, contact transmission, and airborne transmissions and induced COVID-19 within the host and has been spreading across the world (34). And the COVID-19 vaccination is the most effective and cost-effective way to prevent infectious diseases and achieve substantial control of community spread. Therefore, it is critical to systematically estimate Chinese parents' willingness to vaccinate their children against COVID-19 nationally and regionally and to identify predictors of vaccine willingness or vaccine hesitancy.

To our knowledge, this is the first systematic review to investigate the overview of Chinese parents' willingness to vaccinate their children against COVID-19. We found that the pooled rate of parents' willingness to vaccinate their children was 70.0% (95% CI: 62.0–78.0%). Among the study areas, the highest willingness rate was observed in the eastern region (71.0%), while the western region reported the lowest rate (50.0%). In terms of predictors, level of education, history of children's vaccination against influenza, parents' willingness to vaccinate themselves against COVID-19, perceived susceptibility of children infected with COVID-19, parental vaccination hesitancy, and concerns about the safety and efficacy of vaccines were observed to be potentially significant factors of Chinese parents' willingness to vaccinate their children against COVID-19. Our findings are beneficial to provide references for improving future vaccination rates, getting global politics, economics and other aspects back on track and even promoting herd immunity.

We estimated that the pooled rate of parents' willingness to vaccinate their children against COVID-19 was 70.0% (95% CI: 62.0–78.0%), which was lower than the Chinese adult vaccination willingness rate (70.0 vs. 90.6%) (35). This may be explained by the fact that children infected with SARS-CoV-2 usually show mild or no symptoms, which may cause parents to feel less anxious and result in a lower vaccination willingness (17, 36). In contrast, the number of adults who were aware of the necessity of vaccination and were self-vaccinating was increasing. With the development of the COVID-19 vaccine and its widespread use worldwide, WHO surveillance data have shown an increase in the number of COVID-19 infections in unvaccinated children and adolescents (2). In addition, children are an important part of achieving substantial control of community spread (2, 8). Thus, improving COVID-19 vaccination rates for children is beneficial both for protecting children from SARS-CoV-2 infection and preventing other people from contracting the disease by reducing sources of infection and protecting susceptible populations.

A study reported that the coverage rate and full vaccination rate of the first dose of the COVID-19 vaccine in the Chinese population aged 3–17 years reached more than 98 and 95%, respectively (37). This rate is higher than the pooled rate of Chinese parents willing to have their children vaccinated with COVID-19 (70%) found in this meta-analysis. This may be due to subjective bias in parents' willingness to vaccinate their children against COVID-19, which may be influenced by factors such as parents' education, age, and trust in the COVID-19 vaccine and social environment. And children's vaccination rates are also affected by the policy. Therefore, the reported vaccination willingness may not reflect actual vaccination behavior as well. In addition, all the included studies were limited by the dynamic nature of the COVID-19 pandemic; the acceptance of the COVID-19 vaccine is dynamic and changes with legislation and public awareness policies (17).

Among the sociodemographic factors, education level was an effective predictor. Parents with low education levels were more likely to vaccinate their children against COVID-19 than those with high levels of education. However, the result from one recent analysis (17) did not indicate education level as an influencing predictor. This inconsistent finding may be because the sample collected for the previous systematic review had a large majority of highly educated parents, and the large difference in sample size between the two subgroups may have influenced this result. Education level can also influence parental willingness to vaccinate their children against COVID-19 by influencing parental preferences for sources of information about obtaining the COVID-19 vaccine. Among several kinds of official or unofficial information sources, including medical advice, personal beliefs, web/social media, and television, one study (19) suggested that COVID-19 vaccination triggered intensive responses on social media among Chinese parents, as approximately 70% of the participants were sometimes or always exposed to information specific to COVID-19 vaccination on different social media platforms. Although some studies have suggested that interpersonal communication and the dissemination of information on social media exacerbate people's trust in false information, one study demonstrated that unofficial sources are indispensable health information publishers and disseminators during the current pandemic which will help people to increase the trust in the social environment, including trust in the government and medical personnel, and the vaccine, which played an important role in influencing people's conduct in terms of health protection from the perspective of the prevention and control of COVID-19 (16). Thus, social media need to be a powerful instrument to distribute the accuracy and objectivity of information and suggestions on the COVID-19 epidemic. Moreover, just as the Indian government has asked social media companies to carefully regulate the content they display and curb the spread of such misinformation about COVID-19 and its vaccines, and has also raised Internet regulation laws and imposed arrests and severe penalties for violators, the Chinese government departments should reasonably manage the double-edged sword of social media to reduce parents' hesitation about vaccines (34).

We also found that the age of parents was not an effective predictor. This finding was inconsistent with a previous study (17), which reported that older parents were more likely to vaccinate their children against COVID-19 than younger parents. This may be because the data collected for this study had a larger proportion of younger than older parents, whereas the data collected for the previous study had a much larger number of older than younger parents. The difference in the proportion of people in the two subgroups may have led to the inconsistent results.

The association between parental occupation (whether health care-related or not) and vaccination intentions is also controversial. Parents whose careers are related to health care have access to better information about viruses and vaccines, giving them more decision-making tools to avoid falling victim to conspiracy theories and making them more likely to vaccinate their children. However, those parents also frequently expressed concerns about the safety, efficacy, and unknown side effects of newly developed vaccines (29, 38, 39). As one study reported that the social trust, which plays an important role in influencing people's conduct in terms of health protection from the perspective of the prevention and control of COVID-19, is dependent on the information provided by the government, medical authorities, and health-care facilities (16). In addition, a previous study reported that the most trusted source of information about COVID-19 vaccines is the child's doctor or health care provider (40). Therefore, government agencies need to explain the safety, accessibility, side effects, and efficacy of the COVID-19 vaccine in a more professional and scientific way for health care-related parents to release their concerns and increase the trust in the social environment and the vaccine, and pediatric providers also need to communicate about the COVID-19 vaccine used in children during routine office visits to improve immunization rates.

In terms of vaccine factors, parental attitudes toward vaccination and the perceived susceptibility of children infected with COVID-19 were critical to improving COVID-19 vaccination rates for children, as parents are the ones who decide whether to vaccinate their children against this disease. The trust in and acceptance of official and unofficial information about the COVID-19 pandemic and the safety, effectiveness, unknown effects, and side effects of the COVID-19 vaccine are the elements that influence the above two aspects (14, 19). Therefore, as some previous studies have suggested, we should improve the accessibility and convenience of parents to accurate and easily accessible information about the COVID-19 vaccine, and increase the dissemination of information related to COVID-19 (41–43). As reported in previous articles, government and medical workers are trusted sources of information for people with the COVID-19 vaccine (16). Thus, consistent with previous research recommendations, the government cooperating with vaccine development companies and health care workers, should disclose more openly and transparently information about vaccine research, development processes, vaccine safety testing, and disseminate other accurate and reliable information about the COVID-19 vaccine to reduce parental concerns about the safety and efficacy of the COVID-19 vaccine (41, 44).

This systematic review has several limitations. First, the sample size of this survey in the central and western regions was small, and the number of included studies that fulfilled the criteria was relatively inadequate, which restricted the comprehensiveness of the data. Second, the included subgroup analysis only comprised 3 to 7 studies for each grouping component, which had an impact on the study's conclusions. Future studies on the factors that influence parents' willingness to vaccinate their children will be necessary to confirm the findings of this study. Third, the majority of the included studies recruited participants *via* convenience sampling, and the lack of a random sampling procedure may have affected the sample's representativeness. Fourth, the majority of the included studies used online questionnaires or surveys, which may be difficult for those who live in poor conditions, and participants were mostly recruited from immunization clinics, physical examination centers, and other medical facilities or schools. Fifth, the pooled rate of Chinese parents' willingness to vaccinate their children against COVID-19 (70%) is not a constant value and is subjective, unlike the COVID-19 vaccination uptake rate. And it is also influenced by modifiable and unmodifiable factors such as the changes in policy, public acceptance of information about the vaccine, and perceived risk of COVID-19 infection to themselves and their children.

This systematic review has two strengths. First, to our knowledge, the study was the first study that systematically estimate Chinese parents' willingness to vaccinate their children against COVID-19 nationally and regionally and to identify predictors of vaccine willingness or vaccine hesitancy. Second, the results of the meta-analysis may be helpful for decision/policymakers to develop policy on evidence-based research and for the child's doctor or healthcare provider to take the right approach to enhance parental willingness to vaccinate their children against COVID-19, achieve substantial control of community spread, getting global politics, economics and other aspects back on track and even promoting herd immunity.

## Conclusions

In general, Chinese parents were moderately willing to vaccinate their children against COVID-19 (70%). Notably, the education level, parents' willingness to vaccinate themselves against COVID-19, the perceived susceptibility of children infected with COVID-19, parental vaccination hesitancy, concerns about the safety and efficacy of vaccines, and the history of children's vaccination against influenza were all potential factors that may affect vaccination decisions. This study can serve as a theoretical guide for enhancing parental willingness to vaccinate their children against COVID-19, achieving substantial control of community spread, getting global politics, economics and other aspects back on track and even promoting herd immunity.

## Data availability statement

The raw data supporting the conclusions of this article will be made available by the authors, without undue reservation.

## Author contributions

YM generated the idea and designed the study. YM, DC, and YZ conducted the literature screening. YM, YL, and SL conducted the data extraction. YM and YZ conducted the data analysis and interpretation. YM wrote the manuscript and revised the paper with significant editorial contributions and discussion from YZ and JR contributed to the final revision of the paper. All authors have read and agreed to the published version of the manuscript.

## Footnotes

^1^World Health Organization. Coronavirus disease (COVID-19). https://covid19.who.int/ [Accessed June 22, 2022].
