# Supplementary material for: Chinese parents' willingness to vaccinate their children against COVID-19: A systematic review and meta-analysis
Source: Front Public Health. 2022 Dec 15;10:1087295. doi: 10.3389/fpubh.2022.1087295 (PMC9798204; doi:10.3389/fpubh.2022.1087295)
Supplement: Supplementary file 1 [file Table_1.docx]

Supplementary Material

# Supplementary Table

Supplementary Table 1 Methodological quality assessment

| Reference | Criteria | | | | | | | |  |
| --- | --- | --- | --- | --- | --- | --- | --- | --- | --- |
|  | Selection | | | | Comparability | | Outcome | |  |
|  | Representativeness of the sample | Sample size | Non –responders | Ascertainment of exposure/risk factor | The study controls for the most important factor | The study control for any additional factor | Assessment of the outcome | Statistical test | Total score  (10) |
| Ke Chun Zhang，2020 | 1 | 1 | 1 | 1 | 1 | 1 | 1 | 1 | 8 |
| Kezhong A,2021 | 1 | 1 | 0 | 1 | 1 | 1 | 1 | 1 | 7 |
| Ting Li,2022 | 1 | 1 | 0 | 1 | 1 | 1 | 1 | 1 | 7 |
| Mei-Xian Zhang,2021 | 1 | 1 | 1 | 1 | 1 | 1 | 1 | 1 | 8 |
| Qiang Wang,2021 | 1 | 1 | 0 | 2 | 1 | 1 | 1 | 1 | 8 |
| Xiao Wan,2021 | 1 | 1 | 1 | 1 | 1 | 1 | 1 | 1 | 8 |
| Jin Yang,2022 | 1 | 1 | 0 | 1 | 1 | 1 | 1 | 1 | 7 |
| Yucheng Xu,2021 | 1 | 1 | 1 | 1 | 1 | 1 | 1 | 1 | 8 |
| Yulan Lin,2021 | 1 | 1 | 0 | 1 | 1 | 1 | 1 | 1 | 7 |
| Yunyun Xu1，2021 | 1 | 1 | 0 | 1 | 1 | 1 | 1 | 1 | 7 |
| Yehong Zhou，2021 | 1 | 1 | 0 | 1 | 1 | 1 | 1 | 1 | 7 |
| Yu Wu,2021 | 1 | 1 | 1 | 2 | 1 | 1 | 1 | 1 | 9 |
| Lilong  Wu,2021 | 1 | 1 | 0 | 1 | 1 | 1 | 1 | 1 | 7 |

Methodological quality assessment of cross-sectional studies using modified Newcastle‒Ottawa Scale (NOS)

Note: from each item account point. (Accept the study if total score ≥5)

Selection: (Maximum 5 stars)

1) Representativeness of the sample:

a) Truly representative of the average in the target population. * (all subjects or random sampling)

b) Somewhat representative of the average in the target population. * (nonrandom sampling).

c) Selected group of users.

d) No description of the sampling strategy.

2) Sample size:

a) Justified and satisfactory. *

b) Not justified.

3) Non-respondents:

a) Comparability between respondents and non-respondents characteristics is established, and the response rate is satisfactory. *

b) The response rate is unsatisfactory, or the comparability between respondents and non-respondents is unsatisfactory.

c) No description of the response rate or the characteristics of the responders and the non-responders.

4) Ascertainment of the exposure (risk factor):

a) validated measurement tool. **

b) Non-validated measurement tool, but the tool is available or described. *

c) No description of the measurement tool.

Comparability: (Maximum 2 stars)

1) The subjects in different outcome groups are comparable, based on the study design or analysis. Confounding factors are controlled.

a) The study controls for the most important factor (select one). *

b) The study control for any additional factor. *

Outcome: (Maximum 3 stars)

1) Assessment of the outcome:

a) Independent blind assessment. **

b) Record linkage. **

c) Self report. *

d) No description.

2) Statistical test:

a) The statistical test used to analyze the data is clearly described and appropriate, and the measurement of the association is presented, including confidence intervals and the probability level (p value). *

b) The statistical test is not appropriate, not described or incomplete

We have not selected one factor that is the most important for comparability, because the variables are not the same in each study. Thus, the principal factor should be identified for each study.
