# Supplementary material for: Chinese parents' willingness to vaccinate their children against COVID-19: A systematic review and meta-analysis
Source: Front Public Health. 2022 Dec 15;10:1087295. doi: 10.3389/fpubh.2022.1087295 (PMC9798204; doi:10.3389/fpubh.2022.1087295)
Supplement: Supplementary file 2 [file Data_Sheet_1.DOCX]

Supplementary Material

# Supplementary Data

## Appendix 1. Search strategies for traditional database.

We conducted a comprehensive search **PubMed, Cochrane Library, Embase,** and the databases in Chinese, including **CNKI, WanFang, VIP, CBM**, using a combination of Medical Subject Headings and free text including terms related to parents, legal guardians, COVID-19, SARS-CoV-2, COVID-19 vaccines, willingness, intention, vaccination hesitancy, vaccination refusal, acceptance, China, and Chinese. At this time, “AND” and “OR” Boolean operators were employed to integrate the keywords. All related published papers **from December 2019 to June 2022** were identiﬁed and subsequently stored using EndNote X9.

**CNKI**

| **NO.** | **Query** | **Results** |
| --- | --- | --- |
| **#1** | (家长 OR 监护人 OR 父母)[Subject/title] | **\** |
| **#2** | (新冠病毒 OR 新型冠状病毒 OR 新型冠状病毒肺炎 OR 新冠肺炎OR 新冠病毒肺炎 OR COVID-19 OR SARS-CoV-2)[Subject/title] | **\** |
| **#3** | (新冠病毒疫苗 OR 新型冠状病毒疫苗 OR 新型冠状病毒肺炎疫苗 OR 新冠肺炎疫苗 OR 新冠病毒肺炎疫苗 OR COVID-19疫苗 OR SARS-CoV-2疫苗)[Subject/title] | **\** |
| **#4** | (意愿 OR 意向 OR 犹豫 OR 拒绝 OR 接受)[Subject/title] | **\** |
| **#5** | (中国 OR China OR Chinese)[Mainland] | **\** |
| **#6** | #1 AND #2 AND #3 AND #4 AND #5 | **24** |

**VIP**

| **NO.** | **Query** | **Results** |
| --- | --- | --- |
| **#1** | (家长 OR 监护人 OR 父母)[Title/Abstract] | **\** |
| **#2** | (新冠病毒 OR 新型冠状病毒 OR 新型冠状病毒肺炎 OR 新冠肺炎 OR 新冠病毒肺炎 OR COVID-19 OR SARS-CoV-2)[Title/Abstract] | **\** |
| **#3** | (新冠病毒疫苗 OR 新型冠状病毒疫苗 OR 新型冠状病毒肺炎疫苗 OR 新冠肺炎疫苗 OR 新冠病毒肺炎疫苗 OR COVID-19疫苗 OR SARS-CoV-2疫苗)[Title/Abstract] | **\** |
| **#4** | (意愿 OR 意向 OR 犹豫 OR 拒绝 OR 接受)[Title/Abstract] | **\** |
| **#5** | (中国 OR China OR Chinese)[Mainland] | **\** |
| **#6** | #1 AND #2 AND #3 AND #4 AND #5 | **3** |

**WanFang**

| **NO.** | **Query** | **Results** |
| --- | --- | --- |
| **#1** | (家长 OR 监护人 OR 父母)[Title] | **\** |
| **#2** | (新冠病毒 OR 新型冠状病毒 OR 新型冠状病毒肺炎 OR新冠肺炎 OR 新冠病毒肺炎 OR COVID-19 OR SARS-CoV-2)[Title] | **\** |
| **#3** | (新冠病毒疫苗 OR 新型冠状病毒疫苗 OR 新型冠状病毒肺炎疫苗 OR 新冠肺炎疫苗 OR 新冠病毒肺炎疫苗 OR COVID-19疫苗 OR SARS-CoV-2疫苗)[Title] | **\** |
| **#4** | (意愿 OR 意向 OR 犹豫 OR 拒绝 OR 接受)[Title] | **\** |
| **#5** | (中国 OR China OR Chinese)[Mainland] | **\** |
| **#6** | #1 AND #2 AND #3 AND #4 AND #5 | **6** |

**CBM**

| **NO.** | **Query** | **Results** |
| --- | --- | --- |
| **#1** | 法定监护人 [MeSH] OR (家长 OR 父母)[Common Fields :Smart] | **\** |
| **#2** | (新冠病毒 OR 新型冠状病毒 OR 新型冠状病毒肺炎 OR 新冠肺炎 OR 新冠病毒肺炎 OR COVID-19 OR SARS-CoV-2）[Common Fields :Smart] | **\** |
| **#3** | (新冠病毒疫苗 OR 新型冠状病毒疫苗 OR 新型冠状病毒肺炎疫苗 OR 新冠肺炎疫苗 OR 新冠病毒肺炎疫苗 OR COVID-19疫苗 OR SARS-CoV-2疫苗)[Common Fields :Smart] | **\** |
| **#4** | 接种拒绝[MeSH] OR (意愿 OR 意向 OR 犹豫 OR 接受)[Common Fields :Smart] | **\** |
| **#5** | 中国[MeSH] OR (China OR Chinese)[All fields :Smart] | **\** |
| **#6** | #1 AND #2 AND #3 AND #4 AND #5 | **9** |

**Pubmed**

| **NO.** | **Query** | **Results** |
| --- | --- | --- |
| **#1** | (Parents [Mesh] OR (Parent OR Parenthood Status OR Status, Parenthood OR Step-Parents OR Step Parents OR Step-Parent OR Stepparent OR Stepparents OR Parental Age OR Age, Parental OR Ages, Parental OR Parental Ages)[Title/Abstract]) OR (Legal Guardians[Mesh] OR (Guardian, Legal OR Guardians, Legal OR Legal Guardian)[Title/Abstract]) | **\** |
| **#2** | (COVID-19[Mesh] OR (COVID 19 OR SARS-CoV-2 Infection OR Infection, SARS-CoV-2 OR SARS CoV 2 Infection OR SARS-CoV-2 Infections OR 2019 Novel Coronavirus Disease OR 2019 Novel Coronavirus Infection OR 2019-nCoV Disease OR 2019 nCoV Disease OR 2019-nCoV Diseases OR Disease, 2019-nCoV OR COVID-19 Virus Infection OR COVID 19 Virus Infection OR COVID-19 Virus Infections OR Infection, COVID-19 Virus OR Virus Infection, COVID-19 OR Coronavirus Disease 2019 OR Disease 2019, Coronavirus OR Coronavirus Disease-19 OR Coronavirus Disease 19 OR Severe Acute Respiratory Syndrome Coronavirus 2 Infection OR SARS Coronavirus 2 Infection OR COVID-19 Virus Disease OR COVID 19 Virus Disease OR COVID-19 Virus Diseases OR Disease, COVID-19 Virus OR Virus Disease, COVID-19 OR 2019-nCoV Infection OR 2019 nCoV Infection OR 2019-nCoV Infections OR Infection, 2019-nCoV OR COVID19 OR COVID-19 Pandemic OR COVID 19 Pandemic OR Pandemic, COVID-19 OR COVID-19 Pandemics)[Title/Abstract]) OR (SARS-CoV-2[Mesh] OR (SARS Coronavirus 2 OR Coronavirus 2, SARS OR Coronavirus Disease 2019 Virus OR 2019 Novel Coronavirus OR 2019 Novel Coronaviruses OR Coronavirus, 2019 Novel OR Novel Coronavirus, 2019 OR Wuhan Seafood Market Pneumonia Virus OR SARS-CoV-2 Virus OR SARS CoV 2 Virus OR SARS-CoV-2 Viruses OR Virus, SARS-CoV-2 OR 2019-nCoV OR COVID-19 Virus OR COVID 19 Virus OR COVID-19 Viruses OR Virus, COVID-19 OR Wuhan Coronavirus OR Coronavirus, Wuhan OR COVID19 Virus OR COVID19 Viruses OR Virus, COVID19 OR Viruses, COVID19 OR Severe Acute Respiratory Syndrome Coronavirus 2)[Title/Abstract]) | **\** |
| **#3** | COVID-19 Vaccines[Mesh] OR (COVID 19 Vaccines OR Vaccines, COVID-19 OR COVID-19 Virus Vaccines OR COVID 19 Virus Vaccines OR Vaccines, COVID-19 Virus OR Virus Vaccines, COVID-19 OR COVID-19 Virus Vaccine OR COVID 19 Virus Vaccine OR Vaccine, COVID-19 Virus OR Virus Vaccine, COVID-19 OR COVID19 Virus Vaccines OR Vaccines, COVID19 Virus OR Virus Vaccines, COVID19 OR COVID19 Virus Vaccine OR Vaccine, COVID19 Virus OR Virus Vaccine, COVID19 OR COVID19 Vaccines OR Vaccines, COVID19 OR COVID19 Vaccine OR Vaccine, COVID19 OR SARS-CoV-2 Vaccines OR SARS CoV 2 Vaccines OR Vaccines, SARS-CoV-2 OR SARS-CoV-2 Vaccine OR SARS CoV 2 Vaccine OR Vaccine, SARS-CoV-2 OR SARS2 Vaccines OR Vaccines, SARS2 OR SARS2 Vaccine OR Vaccine, SARS2 OR Coronavirus Disease 2019 Vaccines OR Coronavirus Disease 2019 Vaccine OR Coronavirus Disease 2019 Virus Vaccine OR Coronavirus Disease 2019 Virus Vaccines OR Coronavirus Disease-19 Vaccines OR Coronavirus Disease 19 Vaccines OR Vaccines, Coronavirus Disease-19 OR Coronavirus Disease-19 Vaccine OR Coronavirus Disease 19 Vaccine OR Vaccine, Coronavirus Disease-19 OR COVID 19 Vaccine OR Vaccine, COVID 19 OR 2019-nCoV Vaccine OR 2019 nCoV Vaccine OR Vaccine, 2019-nCoV OR 2019 Novel Coronavirus Vaccines OR 2019 Novel Coronavirus Vaccine OR 2019-nCoV Vaccines OR 2019 nCoV Vaccines OR Vaccines, 2019-nCoV OR COVID-19 Vaccine OR Vaccine, COVID-19 OR SARS Coronavirus 2 Vaccines )[Title/Abstract] | **\** |
| **#4** | (willingness[Title/Abstract]) OR (Intention[Mesh] OR Intentions[Title/Abstract]) OR (Vaccination Hesitancy[Mesh] OR (Hesitancy, Vaccination OR Vaccination Hesitancies OR Vaccine Hesitancy OR Hesitancy, Vaccine OR Vaccine Hesitancies)[Title/Abstract]) OR (Vaccination Refusal[Mesh] OR (Refusal, Vaccination OR Refusals, Vaccination OR Vaccination Refusals OR Vaccine Refusal OR Refusal, Vaccine OR Refusals, Vaccine OR Vaccine Refusals OR Vaccine Delay OR Delay, Vaccine OR Delays, Vaccine OR Vaccine Delays OR Vaccination Delay OR Delay, Vaccination OR Delays, Vaccination OR Vaccination Delays)[Title/Abstract]) OR (acceptance[Title/Abstract]) | **\** |
| **#5** | (China [Mesh] OR (People's Republic of China OR Mainland China OR Sinkiang OR Inner Mongolia OR Manchuria)) OR Chinese | **\** |
| **#6** | #1 AND #2 AND #3 AND #4 AND #5 | **17** |

**Cochrane Library**

| **NO.** | **Query** | **Results** |
| --- | --- | --- |
| **#1** | Parenting [Mesh] OR (Parent OR Parenthood Status OR Status Parenthood OR Step-Parents OR Step Parents OR Step-Parent OR Stepparent OR Stepparents OR Parental Age OR Age Parental OR Ages Parental OR Parental Ages):ti,ab,kw OR Legal Guardians[Mesh] OR (Guardian, Legal OR Guardians, Legal OR Legal Guardian):ti,ab,kw | **23369** |
| **#2** | **COVID-19**[Mesh] OR (**COVID 19 OR 2019 Novel Coronavirus Disease OR 2019 Novel Coronavirus Infection OR COVID-19 Virus Infection OR COVID 19 Virus Infection OR COVID-19 Virus Infections OR Infection, COVID-19 Virus OR Virus Infection, COVID-19 OR Coronavirus Disease 2019 OR Disease 2019, Coronavirus OR Coronavirus Disease-19 OR Coronavirus Disease 19 OR Severe Acute Respiratory Syndrome Coronavirus 2 Infection OR SARS Coronavirus 2 Infection OR COVID-19 Virus Disease OR COVID 19 Virus Disease OR COVID-19 Virus Diseases OR Disease, COVID-19 Virus OR Virus Disease, COVID-19 OR COVID-19 Pandemic OR COVID 19 Pandemic OR Pandemic, COVID-19 OR COVID-19 Pandemics OR SARS-CoV-2 Infection OR Infection, SARS-CoV-2 OR SARS CoV 2 Infection OR SARS-CoV-2 Infections OR 2019 nCoV Disease OR 2019 nCoV Infection):ti,ab,kw OR SARS-CoV-2**[Mesh] OR (**SARS Coronavirus 2 OR Coronavirus 2, SARS OR Coronavirus Disease 2019 Virus OR 2019 Novel Coronavirus OR 2019 Novel Coronaviruses OR Coronavirus, 2019 Novel OR Novel Coronavirus, 2019 OR Wuhan Seafood Market Pneumonia Virus OR COVID-19 Virus OR COVID 19 Virus OR COVID-19 Viruses OR Virus, COVID-19 OR Wuhan Coronavirus OR Coronavirus, Wuhan OR COVID 19 Virus OR COVID 19 Viruses OR Virus, COVID 19 OR Viruses, COVID 19 OR Severe Acute Respiratory Syndrome Coronavirus 2 OR SARS-CoV-2 Virus OR SARS CoV 2 Virus OR SARS-CoV-2 Viruses OR Virus, SARS-CoV-2):ti,ab,kw** | 10821 |
| **#3** | **COVID-19 Vaccines**[Mesh] OR **COVID 19 Vaccines OR Vaccines, COVID-19 OR COVID-19 Virus Vaccines OR COVID 19 Virus Vaccines OR Vaccines, COVID-19 Virus OR Virus Vaccines, COVID-19 OR COVID-19 Virus Vaccine OR COVID 19 Virus Vaccine OR Vaccine, COVID-19 Virus OR Virus Vaccine, COVID-19 OR COVID 19 Virus Vaccines OR Vaccines, COVID 19 Virus OR Virus Vaccines, COVID 19 OR COVID 19 Virus Vaccine OR Vaccine, COVID 19 Virus OR Virus Vaccine, COVID 19 OR COVID 19 Vaccines OR Vaccines, COVID 19 OR COVID 19 Vaccine OR Vaccine, COVID 19 OR SARS-CoV-2 Vaccines OR SARS CoV 2 Vaccines OR Vaccines, SARS-CoV-2 OR SARS-CoV-2 Vaccine OR SARS CoV 2 Vaccine OR Vaccine, SARS-CoV-2 OR SARS2 Vaccines OR Vaccines, SARS2 OR SARS2 Vaccine OR Vaccine, SARS2 OR Coronavirus Disease 2019 Vaccines OR Coronavirus Disease 2019 Vaccine OR Coronavirus Disease 2019 Virus Vaccine OR Coronavirus Disease 2019 Virus Vaccines OR Coronavirus Disease-19 Vaccines OR Coronavirus Disease 19 Vaccines OR Vaccines, Coronavirus Disease-19 OR Coronavirus Disease-19 Vaccine OR Coronavirus Disease 19 Vaccine OR Vaccine, Coronavirus Disease-19 OR COVID 19 Vaccine OR Vaccine, COVID 19 OR 2019 nCoV Vaccine OR 2019 Novel Coronavirus Vaccines OR 2019 Novel Coronavirus Vaccine OR 2019 nCoV Vaccines OR COVID-19 Vaccine OR Vaccine, COVID-19 OR SARS Coronavirus 2 Vaccines):ti,ab,kw** | **1200** |
| **#4** | **(willingness):ti,ab,kw OR (Intention**[Mesh] OR **(Intentions):ti,ab,kw) OR Vaccination Hesitancy**[Mesh] OR (Hesitancy, Vaccination OR Vaccination Hesitancies OR Vaccine Hesitancy OR Hesitancy, Vaccine OR Vaccine Hesitancies OR Vaccine Delay OR Delay, Vaccine OR Delays, Vaccine OR Vaccine Delays OR Vaccination Delay OR Delay, Vaccination OR Delays, Vaccination OR Vaccination Delays)**:ti,ab,kw OR (Vaccination Refusal**[Mesh] **OR (Refusal, Vaccination OR Refusals, Vaccination OR Vaccination Refusals OR Vaccine Refusal OR Refusal, Vaccine OR Refusals, Vaccine OR Vaccine Refusals):ti,ab,kw) OR (acceptance):ti,ab,kw** | **76678** |
| **#5** | (China [Mesh] OR (People's Republic of China OR Mainland China OR Sinkiang OR Inner Mongolia OR Manchuria)) OR Chinese | **69305** |
| **#6** | #1 AND #2 AND #3 AND #4 AND #5 | 0 |

**EMBACE**

| **NO.** | **Query** | **Results** |
| --- | --- | --- |
| **#1** | ('parent'/exp OR (Parent OR Parenthood Status OR Status, Parenthood OR Step-Parents OR Step Parents OR Step-Parent OR Stepparent OR Stepparents OR Parental Age OR Age, Parental OR Ages, Parental OR Parental Ages): ab,ti) OR ('legal guardian'/exp OR (Guardian, Legal OR Guardians, Legal OR Legal Guardian):ab,ti) | 414439 |
| **#2** | ('coronavirus disease 2019'/exp OR (COVID 19 OR SARS-CoV-2 Infection OR Infection, SARS-CoV-2 OR SARS CoV 2 Infection OR SARS-CoV-2 Infections OR 2019 Novel Coronavirus Disease OR 2019 Novel Coronavirus Infection OR 2019-nCoV Disease OR 2019 nCoV Disease OR 2019-nCoV Diseases OR Disease, 2019-nCoV OR COVID-19 Virus Infection OR COVID 19 Virus Infection OR COVID-19 Virus Infections OR Infection, COVID-19 Virus OR Virus Infection, COVID-19 OR Coronavirus Disease 2019 OR Disease 2019, Coronavirus OR Coronavirus Disease-19 OR Coronavirus Disease 19 OR Severe Acute Respiratory Syndrome Coronavirus 2 Infection OR SARS Coronavirus 2 Infection OR COVID-19 Virus Disease OR COVID 19 Virus Disease OR COVID-19 Virus Diseases OR Disease, COVID-19 Virus OR Virus Disease, COVID-19 OR 2019-nCoV Infection OR 2019 nCoV Infection OR 2019-nCoV Infections OR Infection, 2019-nCoV OR COVID19 OR COVID-19 Pandemic OR COVID 19 Pandemic OR Pandemic, COVID-19 OR COVID-19 Pandemics):ab,ti) OR ('severe acute respiratory syndrome coronavirus 2'/exp OR (SARS Coronavirus 2 OR Coronavirus 2, SARS OR Coronavirus Disease 2019 Virus OR 2019 Novel Coronavirus OR 2019 Novel Coronaviruses OR Coronavirus, 2019 Novel OR Novel Coronavirus, 2019 OR Wuhan Seafood Market Pneumonia Virus OR SARS-CoV-2 Virus OR SARS CoV 2 Virus OR SARS-CoV-2 Viruses OR Virus, SARS-CoV-2 OR 2019-nCoV OR COVID-19 Virus OR COVID 19 Virus OR COVID-19 Viruses OR Virus, COVID-19 OR Wuhan Coronavirus OR Coronavirus, Wuhan OR COVID19 Virus OR COVID19 Viruses OR Virus, COVID19 OR Viruses, COVID19 OR Severe Acute Respiratory Syndrome Coronavirus 2):ab,ti) | 286623 |
| **#3** | 'sars-cov-2 vaccine'/exp OR (COVID 19 Vaccines OR Vaccines, COVID-19 OR COVID-19 Virus Vaccines OR COVID 19 Virus Vaccines OR Vaccines, COVID-19 Virus OR Virus Vaccines, COVID-19 OR COVID-19 Virus Vaccine OR COVID 19 Virus Vaccine OR Vaccine, COVID-19 Virus OR Virus Vaccine, COVID-19 OR COVID19 Virus Vaccines OR Vaccines, COVID19 Virus OR Virus Vaccines, COVID19 OR COVID19 Virus Vaccine OR Vaccine, COVID19 Virus OR Virus Vaccine, COVID19 OR COVID19 Vaccines OR Vaccines, COVID19 OR COVID19 Vaccine OR Vaccine, COVID19 OR SARS-CoV-2 Vaccines OR SARS CoV 2 Vaccines OR Vaccines, SARS-CoV-2 OR SARS-CoV-2 Vaccine OR SARS CoV 2 Vaccine OR Vaccine, SARS-CoV-2 OR SARS2 Vaccines OR Vaccines, SARS2 OR SARS2 Vaccine OR Vaccine, SARS2 OR Coronavirus Disease 2019 Vaccines OR Coronavirus Disease 2019 Vaccine OR Coronavirus Disease 2019 Virus Vaccine OR Coronavirus Disease 2019 Virus Vaccines OR Coronavirus Disease-19 Vaccines OR Coronavirus Disease 19 Vaccines OR Vaccines, Coronavirus Disease-19 OR Coronavirus Disease-19 Vaccine OR Coronavirus Disease 19 Vaccine OR Vaccine, Coronavirus Disease-19 OR COVID 19 Vaccine OR Vaccine, COVID 19 OR 2019-nCoV Vaccine OR 2019 nCoV Vaccine OR Vaccine, 2019-nCoV OR 2019 Novel Coronavirus Vaccines OR 2019 Novel Coronavirus Vaccine OR 2019-nCoV Vaccines OR 2019 nCoV Vaccines OR Vaccines, 2019-nCoV OR COVID-19 Vaccine OR Vaccine, COVID-19 OR SARS Coronavirus 2 Vaccines):ab,ti | 21245 |
| **#4** | 'willingness'/exp OR ((intention OR intentions):ab,ti) OR ('vaccine hesitancy'/exp OR (Hesitancy, Vaccination OR Vaccination Hesitancies OR Vaccine Hesitancy OR Hesitancy, Vaccine OR Vaccine Hesitancies OR Vaccine Delay OR Delay, Vaccine OR Delays, Vaccine OR Vaccine Delays OR Vaccination Delay OR Delay, Vaccination OR Delays, Vaccination OR Vaccination Delays):ab,ti) OR ('vaccination refusal'/exp OR (Refusal, Vaccination OR Refusals, Vaccination OR Vaccination Refusals OR Vaccine Refusal OR Refusal, Vaccine OR Refusals, Vaccine OR Vaccine Refusals):ab,ti) OR 'acceptance'/exp | 106890 |
| **#5** | ('china'/exp OR (People's Republic of China OR Mainland China OR Sinkiang OR Inner Mongolia OR Manchuria)) OR Chinese | 364252 |
| **#6** | **#1 AND #2 AND #3 AND #4 AND #5** | **9** |

## Appendix 2：Explanation of extracted information

Gender of parent: defined as the biological sex of the parents of children aged 3 to 17 years, which can be classified as male or female.

Level of education: defined as the highest level of education attained by the parents, which can be separated into two groups: high school and below education and university and above education.

Marital status: defined as the marital status of parents of children aged 3 to 17, they can be divided into a two-parent family group and a single-parent family group.

Health care-related occupations: Based on whether their occupations were linked to health care or not, the parents were separated into two groups: health care-related and non-health care related.

The number of willing participants: defined as the number of parents who were willing to vaccinate their children.

Parental perceived susceptibility of children infected with COVID-19: defined as whether the parent believes the child is at high risk of infecting COVID-19.

History of children's influenza vaccination: defined as whether children have a history of influenza vaccination.

Parental perceived efficacy and safety of COVID-19 vaccines: defined as whether parents believe the COVID-19 vaccine is safe and effective.

Parental willingness to vaccinate themselves against COVID-19: defined as parents' self-willingness to receive the COVID-19 vaccine.

Parental vaccination hesitancy: characterized as a parent's hesitation to vaccinate their children with the COVID-19 vaccine.
